# Measuring prostate-specific quality of life in prostate cancer patients scheduled for radiotherapy or radical prostatectomy and reference men in Germany and Canada using the Patient Oriented Prostate Utility Scale-Psychometric (PORPUS-P)

**DOI:** 10.1186/1471-2407-9-295

**Published:** 2009-08-23

**Authors:** Annika Waldmann, Volker Rohde, Karen Bremner, Murray Krahn, Thomas Kuechler, Alexander Katalinic

**Affiliations:** 1 Institute of Cancer Epidemiology, University Luebeck, Beckergrube 43-47, 23552 Luebeck, Germany; 2 University Hospital of Giessen, Dept. of Urology and Pediatric Urology, Rudolf-Buchheim-Straße 7, 35392 Giessen, Germany; 3 Toronto General Hospital, University Health Network, EN13-241, 200 Elizabeth Street, Toronto, Ontario, M5G 2C4, Canada; 4 Toronto Health Economics and Technology Assessment (THETA) Collaborative, University of Toronto, 144 College Street, Room 600, Toronto, Ontario M5S 3M2, Canada; 5 Department of General and Thoracic Surgery, Reference Center Quality of Life in Oncology, University Hospital of Schleswig-Holstein, Arnold-Heller-Straße 7, 24105 Kiel, Germany

## Abstract

**Background:**

The PORPUS-P is a short questionnaire for measuring prostate-specific quality of life (QoL), which was designed in Canada for use in prostate cancer (PC) patients. We aimed to generate a German version and compare PORPUS-P scores of German reference men from the general population, and German and Canadian patients with newly diagnosed PC who were scheduled to receive radical prostatectomy (RP) or radiotherapy (RT).

**Methods:**

The study sample consisted of 988 reference men, 121 German and 66 Canadian PC patients scheduled for RT, and 371 German and 68 Canadian PC patients scheduled for RP. All men completed the PORPUS-P (German postal questionnaire, Canada personal interview). Data were gathered from PC patients before the start of therapy.

**Results:**

Canadian patients were better educated than the German patients, and fewer were retired. Patients scheduled to receive RT were older and more were retired. German RT patients had lower D'Amico risk scores and pre-treatment Gleason scores than RP patients, and Canadian RT patients had higher pre-treatment PSA than RP patients. Urinary and sexual dysfunction were seen in PC patients (especially RT patients), but were also common in the German reference men. Crude mean PORPUS-P scores differed statistically significant between German RT and RP and Canadian RP and RT patients, with RT patients having higher QoL scores. The differences in age-adjusted mean PORPUS-P scores between reference men and RP patients were not clinically significant, while RT patients had (clinically) significantly lower scores than the reference men.

**Conclusion:**

The German translation of the PORPUS-P appears to be a short and feasible tool for assessing prostate-specific QoL. Although we found a similar response pattern, Canadian and German PC patients scheduled to receive RT or RP rated their pre-treatment quality of life on different levels, which reveals the need for national reference data. Problems in several QoL domains exist before treatment, and differ between PC patients scheduled for RT and RP.

## Background

Prostate cancer (PC) is one of the leading malignancies worldwide, with 543,000 new cases each year [1]. In Germany, tumours of the prostate gland account for about 25% of all malignant diseases (i.e. 58,000 incident cases each year) [2]. It is estimated that in 2008, 24,700 men in Canada were diagnosed with PC (28.4% of all neoplasms in males). Today, 5-year survival rate is > 80% (compared with ~60% in 1970). The increase is mainly due to systematic use of PSA screening and optimized therapy regimens [2,3]. Depending on age and extent of disease different modes of therapies can be used. Patients aged 75 years or more are often not eligible for radiotherapy (RT) or radical prostatectomy (RP), but are treated with androgen deprivation therapy (ADT). In patients with a life expectancy of more than 10 years curative treatment is intended [4]. D'Amico low risk patients [5] can choose between RP and RT (either external beam (EB), seed implantation (BRT) without removal of seeds, or a combination of both of EB and BRT) [4].

However, radiation is frequently used – especially in patients with intermediate or high D'Amico risk score, and a high degree of co-morbidity. Both therapies have a similar impact on survival, but a different impact on quality of life (QoL). The most common late complications of radical prostatectomy are erectile dysfunction (ED; defined as the persistent inability to achieve or maintain an erection sufficient for satisfactory sexual performance [6]), urinary incontinence, inguinal hernia, and urethral stricture. The main adverse side effects of radiation therapy are related to injury to the microvasculature of the bladder, rectum, striated sphincter muscle, and urethra. Approximately half of patients develop ED after radiotherapy for prostate cancer [2]. After radical prostatectomy the long-term impotence rate is approximately 40–75% [7].

Many of these symptoms are also found in the general population. In the United States more than 30 million men have ED and in Germany the age-adjusted ED prevalence rates are estimated to be between 18 and 48% [8]. A 2003 survey of 4,539 Canadian men aged 40 years and older and involved in a heterosexual relationship found that 34% of respondents had ED [9]. Moreover, a study in 2,498 participants demonstrated that urinary incontinence is also frequent in the general population. Overall, 26.3% of women and 5.0% of men reported episodes of urinary incontinence during the past 4 weeks. Prevalence rates increased with age in both sexes [10].

Both ED and urinary incontinence influence activities of daily life, social life, role function, and overall QoL. In the study of Temml and colleagues 65.7% of women and 58.3% of men stated that their QoL was affected by their incontinence [10]. This illustrates the need for QoL instruments that include sexual, urinary and bowel dysfunction. This is particularly important in assessing QoL in PC patients. Unlike most generic QoL questionnaires, the Patient Oriented Prostate Utility Scale (PORPUS) takes the above mentioned key determinants of QoL into account. The PORPUS was designed to be a utility instrument (PORPUS-U), but it can also be used as a psychometric instrument (PORPUS-P) [11,12].

In this paper we present the German version of the PORPUS-P and data from three different groups: a German reference population, German patients with newly-diagnosed PC, and Canadian patients with newly-diagnosed PC.

## Methods

### San BKK-study (Reference data for German males)

A population-based sample of 3,000 men was randomly selected from the computer records of a German statutory health insurance company (Sancura BKK). No medical exclusion or inclusion criteria were defined, as we intended to investigate health-related QoL in the general population. The only exclusion criterion was an age less than 45 or greater than 75 years [13]. First, an introductory letter was sent to the men, describing the study and its aims (i.e. measuring prostate-specific QoL in the general population). After 14 days the study questionnaire, which included questions on sociodemographics and QoL (PORPUS [11,12] and other QoL measures), was mailed with a pre-stamped return envelope. The mailing was conducted during March and April 2004. During this time period Sancura BKK personnel were available by telephone in case of questions about the questionnaires.

All 1,150 males who returned their completed questionnaire within four weeks were in our reference sample of German males.

We followed a strict anonymization protocol by which study identification numbers were kept separate from personal data such as name and address. In order to provide a high level of anonymity, the mailing was carried out by a neutral private company. Flagging of questionnaires with an identification number was not allowed for data protection reasons. Furthermore, completed questionnaires had to be sent directly to the study centre and not to the neutral private company. Therefore a non-responder analysis was not possible for the San BKK-study.

### The ProCaSP study (German men with newly diagnosed prostate cancer)

Data from men with newly-diagnosed PC were obtained within the prospective cohort study *ProCaSP*. In ten German hospitals 529 patients with PC were recruited, of which 395 were scheduled to have a radical prostatectomy (RP) and 134 were scheduled for radiotherapy (RT) (recruitment period: April 2002 to October 2006).

Exclusion criteria were an age greater than 75 years, the presence of metastasis and/or other primary tumours, a psychiatric diagnosis recorded on the clinic chart, and a history of cognitive impairment. Furthermore, patients were excluded if they were not fluent in German.

All patients received a clinical examination, were asked for co-morbidities and personal case history (information was documented on a standardized case report form) and completed self-administered questionnaires regarding sociodemographic data and QoL (amongst others: PORPUS [11,12]) before they received RT or RP. During the clinic visits trained, skilled personnel were available in case of questions about the questionnaires; afterwards a telephone hotline was implemented.

Data from the clinical investigation (pre-treatment PSA, biopsy Gleason score and clinical stage) were used to compute the D'Amico risk score [5], which is used to estimate the biologic aggressiveness of prostate cancers and to estimate the progression-free survival.

### Canadian Study

Patients diagnosed with clinically localized PC within the previous six months were recruited from ambulatory care clinics of urologists and radiation oncologists at the Princess Margaret Hospital, a regional cancer centre and hospital associated with the University Health Network in Toronto, Canada. Patients were scheduled to receive RP, or external beam radiation therapy or brachytherapy (RT), with or without neo-adjuvant androgen deprivation therapy, for the first time. Patients were excluded if they were not fluent in English, had a documented history of cognitive impairment, or had a psychiatric diagnosis recorded on the clinic chart.

Consenting patients completed the PORPUS and other QoL measures at a personal interview. Demographic and clinical information was collected by self-report and chart review, and included age, marital status, employment, living arrangements, the three most recent PSA levels, tumour stage, androgen deprivation therapy, and co-morbid diseases required to calculate Charlson co-morbidity scores [14].

### Ethics and consent

The German study protocols were approved by the ethics committee of the Giessen university hospital, Germany.

The Canadian study protocol was approved by the ethics review committee of the University Health Network, Toronto.

All persons participated voluntarily and gave written informed consent.

### PORPUS

The PORPUS [11] is a 10-item health state classification system. Each item describes a QoL domain that is relevant to PC. These domains were identified through an iterative process involving interviews with patients and clinical experts, and include five broad QoL domains (pain, energy, emotional well-being, social support, relationship with medical doctor) and five PC-specific domains (sexual function and desire, urinary frequency and incontinence, bowel function). Within each domain or item, there are four to six text descriptions representing a range of symptom severity. For example, for urinary leakage, the worst level is "Require a clamp, catheter, or collecting bag because of leaking urine or poor bladder control" and the best level is "Never, under any circumstances, leak urine or lose bladder control".

The PORPUS may be used as a profile, disease-specific, non-preference based instrument to compute a QoL measure that we call the PORPUS-P [11,12]. The PORPUS can also aid in direct utility assessment when it is administered by a trained interviewer using a script (available on request), 4 marker states, and visual props for Rating Scale and Standard Gamble elicitation [11]. Patients first describe their current health by selecting one level from each attribute of the PORPUS-P. This health state description is placed on a colour-coded card and patients rank it, and 4 marker states (mild impairment, severe impairment, full health, and death), in order of preference. Patients' utilities for their own health state, and the mild and severe impairment marker states, are obtained using the Rating Scale (PORPUS-U_RS_) and Standard Gamble (PORPUS-U_SG_), following the script. This prompted method of utility assessment has face, content, and construct validity, and good test-retest reliability [11,12,15,16], but it is time-consuming and requires a trained interviewer and visual props. Recently, utility weights for PORPUS health states were developed, using direct utility assessment in 234 prostate cancer patients in Canada and a multi-attribute utility function [Tomlinson G, Bremner K, Ritvo P, Naglie G, Krahn M: Development and validation of a utility weighting function for the PORPUS – Patient Oriented Prostate Utility Scale, submitted]. Thus, the PORPUS can also be used as an indirect, disease-specific utility instrument.

### German Version of the PORPUS

Following the EORTC guidelines for translation procedures [17], we used the forward-backward-translation method to generate the German version of the original English-Canadian PORPUS questionnaire [11,12,18]. The translation process began with forward translation into German conducted by a German native speaker with a high level of fluency in English. Backward translation was conducted independently of the first translator by a Canadian who was fluent in German. This process was repeated until a satisfactory translation was obtained.

The item ''Emotional well-being" is a 5-point item in the original Canadian PORPUS-P. Because there is no adequate German translation of "quite a bit" in contrast to "moderate" and "little" the German version of this item was constructed as a 4-point item (see additional file 1). Therefore, we were unable to score the PORPUS as an utility instrument.

### Scoring procedure

According to the scoring manual the scores for each item range from 0 to 3 (in case of 4 point scales), 4 or 5 (5 or 6 point scales respectively) with high values indicating a high level of deterioration or symptomatology. After rescaling the data so that the range within each item is equivalent (i.e. dividing each item by the number of possible answer categories) all values are summed and multiplied by 10 and by the number of required responses divided by the number of completed items. The resulting value has to be subtracted from 100 to calculate the PORPUS-P-score (see Formula 1).

The resulting PORPUS-P-score ranges from 0 to 100 with high values indicating high QoL. At least 8 out of 10 items have to be completed for a PORPUS-P score to be computed.

N_1 _is the number of responses required (i.e., 10)

N_2 _is the number of non-missing responses

*S *are the non-missing responses

Formula 1: Equation for computing PORPUS-P scores

### Statistical methods

SPSS 15.0 (SPSS version 15.0 for Windows, SPSS Inc. Chicago, Illinois, USA; 1989–2006) was used to analyse the German data.

SAS version 9.1 (SAS 9.1 for Windows, SAS Inc. Cary, N.C., USA; 2002–2003) was used for all analyses of the Canadian data.

We used descriptive statistics (means, standard deviations (SD), median and 5^th ^and 95^th ^percentiles), analysis of variance, and chi-square tests to describe and compare the baseline characteristics of the two patient groups (RP, RT) within each country. Distribution of PORPUS-P scores was checked visually (histograms) and using Kolmogorov-Smirnov tests.

Spearman correlation coefficient is reported for the association between age categories and PORPUS-P scores. For interpretation of correlation coefficients the following ranges were chosen: correlation coefficients ranging from zero to 0.2 were considered as indicating a very low association, 0.2 to 0.5 = low association, 0.5 to 0.7 = moderate association, greater than 0.7 = high association. Two-sided p-values ≤ 0.05 were considered significant.

In order to check whether the fact that there were missing values in the German studies but not in the Canadian study biased the PORPUS-P results or not, best-case and worst-case analyses were performed. For this purpose missing values were replaced with either the value indicating the level with no problems (i.e. "zero"), or the value indicating the level with the most severe problems (depending on the PORPUS-item; score "three", "four", or "five"), respectively.

Indirect age-standardization was used to generate age-adjusted means for the PC patients (reference age structure = age structure in German reference males). During this process the age distributions of the different subgroups (German and Canadian RT and RP patients) are standardized to the age structure observed in German reference males. Because young German patients scheduled to receive radiotherapy were not present in the data set, the age-group 45 to 49 years had to be excluded for the indirect age-standardization.

The resulting adjusted mean is the mean that would have been observed if the patient groups would have had the same age structure as the reference men.

## Results

### Study cohort 1: German reference males

Due to missing or implausible information on age (n = 16), education (n = 20), working status (n = 2), marital status (n = 6), or one of the items needed to compute scores on the QoL instruments (PORPUS-P or other QoL instruments that were included in the questionnaire; n = 125), 162 males had to be excluded from the analysis. The total sample consisted of 988 men from the German general population. Baseline data is shown in Tables 1, 2, 3, 4.

**Table 1:** Age distribution of the study samples (N; percentages in parentheses)

	German reference men [n = 988]	German PC patients with scheduled radiotherapy [n = 121]	German PC patients with scheduled radical prostatectomy [n = 371]	p-values for differences between German RT and RP patients	Canadian PC patients with scheduled radiotherapy [n = 66]	Canadian PC patients with scheduled radical prostatectomy [n = 68]	p-values for differences between Canadian RT and RP patients
Age [years] Mean ± SD	56 ± 7.6	67 ± 5.1	63 ± 6.2	p < 0.001*	68 ± 6.3	60 ± 6.3	p < 0.001*

Age category [years]				p < 0.001#			p < 0.001#
45–49	253 (26)	0 (0)	13 (4)		1 (2)	1 (1)	
50–54	239 (24)	2 (2)	31 (8)		2 (3)	14 (20)	
55–59	162 (16)	10 (8)	57 (15)		4 (6)	18 (26)	
60–64	166 (17)	26 (22)	117 (32)		9 (14)	17 (25)	
65–69	127 (13)	43 (36)	115 (31)		21 (20)	11 (16)	
70+	41 (4)	40 (33)	38 (10)		29 (44)	7 (10)	

* analysis of variance # chi-square tests

**Table 2:** Characteristics of the study samples (N; percentages in parentheses)

	German reference men [n = 988]	German PC patients with scheduled radiotherapy [n = 121]	German PC patients with scheduled radical prostatectomy [n = 371]	p-values for differences between German RT and RP patients	Canadian PC patients with scheduled radiotherapy [n = 66]	Canadian PC patients with scheduled radical prostatectomy [n = 68]	p-values for differences between Canadian RT and RP patients
Education				p = 0.980#			p = 0.50#
high school or less (up to 11–12 yrs)	768 (78)	84 (69)	258 (69)		23 (35)	20 (29)	
college or university (12 yrs or more)	220 (22)	37 (31)	113 (31)		43 (65)	48 (71)	

Part-/fulltime workers	588 (60)	16 (13)	135 (36)	p =< 0.001#	22 (33)	47 (69)	p < 0.001#

Married or common law	894 (91)	111 (92)	350 (94)	p = 0.306#	59 (89)	54 (79)	p = 0.112#

Living alone	73 (7)	9 (7)	22 (6)	p = 0.548#	9 (9)	7 (10)	p = 0.81#

# chi-square tests

**Table 3:** Clinical characteristics of the study samples (N; percentages in parentheses)

	German reference men [n = 988]	German PC patients with scheduled radiotherapy [n = 121]	German PC patients with scheduled radical prostatectomy [n = 371]	p-values for differences between German RT and RP patients	Canadian PC patients with scheduled radiotherapy [n = 66]	Canadian PC patients with scheduled radical prostatectomy [n = 68]	p-values for differences between Canadian RT and RP patients
D'Amico Risk Score	Not			p < 0.001#			
Low		39 (32.2)	24 (6.5)				
Intermediate		39 (32.2)	46 (12.4)				
High	applicable	43 (35.5)	301 (81.1)		-	-	

Gleason score (pre-treatment) Mean ± SD	Not applicable	6.13 ± 1.33	6.35 ± 1.21	p < 0.096*	-	-	

Highest recorded pre-treatment PSA [ng/ml] (mean ± SD)	Not applicable	13.7 ± 17.3	10.2 ± 17.9	p = 0.066*	14.3 ± 14.6	8.81 ± 6.9	p = 0.006*

Gleason score and D'Amico risk score were not assessed in the Canadian study

* analysis of variance # chi-square tests

**Table 4:** Tumor stage at diagnosis of the study samples (N; percentages in parentheses)

	German reference men [n = 988]	German PC patients with scheduled radiotherapy [n = 121]	German PC patients with scheduled radical prostatectomy [n = 371]	p-values for differences between German RT and RP patients	Canadian PC patients with scheduled radiotherapy [n = 66]	Canadian PC patients with scheduled radical prostatectomy [n = 68]	p-values for differences between Canadian RT and RP patients
Tumour stage at diagnosis	Not applicable			p < 0.001#			p = 0.008#
T1		28 (23.1)	2 (0.5)		18 (27)	21 (31)	
T2		34 (28.1)	239 (64.4)		29 (44)	30 (44)	
T3		20 (16.5)	122 (32.9)		14 (21)	1 (1.5)	
T4		0	0		1 (1.5)	0	
missing		39 (32.2)	8 (2.2)		4 (6)	16 (23.5)	

# chi-square tests

The 162 excluded men (with valid data) were – compared to men of our reference sample – a little older (mean age: 58.5 ± 8.1 years; p < 0.001), more likely to be retired (48.7%; p < 0.001), less educated (< = 9 years schooling: 69.7%; p = 0.053), and reported lower health related quality of life on the PORPUS-P (crude mean and standard deviation: 78.5 ± 15.6 vs. 83.5 ± 12.1; p < 0.001).

### Study cohort 2: German Patients with prostate cancer

In total, 529 patients with newly-diagnosed PC were recruited and completed a baseline questionnaire. A subsample of 496 provided valid data to compute baseline PORPUS-P (33 had more than two missing answers, thus no PORPUS-P score could be computed). A further four patients were excluded from the final analysis due to missing sociodemographic (n = 2) or clinical data (Gleason score; n = 2). Therefore, our final cohort consisted of 492 men with PC with complete baseline questionnaires, of whom 121 were scheduled to receive RT and 371 were scheduled to receive radical prostatectomy. Systematic differences in age, sociodemographics, D'Amico risk scores, and therapy between persons included in our analyses (n = 492) and those who were excluded (n = 37) were not observed (*data not shown*).

Among the 121 RT patients, 39 patients (32.2%) were scheduled to receive BRT and 33 (27.3%) were scheduled to receive a combination of EB and BRT. BRT patients did not differ from the other RT patients in age (BRT: 65.7 ± 5.1 years, EB/BRT: 66.4 ± 5.4 years, EB: 67.6 ± 4.9 years; p = 0.188) or pre-treatment PSA (BRT: 11.4 ± 15.8 ng/ml, EB/BRT: 16.7 ± 13.3 ng/ml, EB: 13.5 ± 20.4 ng/ml; p = 0.440). However, patients with BRT or EB/BRT had lower Gleason scores (BRT: 5.6 ± 1.1, EB/BRT: 6.2 ± 1.7, EB: 6.5 ± 1.1; p = 0.01) and less frequent small tumours (BRT: 14.3% T1, EB/BRT: 3.6% T1, EB: 55.3% T1, p < 0.001).

Fewer patients scheduled to receive RP were on androgen deprivation therapy (ADT; 1.9%) than RT patients (40.5%, 49 cases). Of the latter, 12 were scheduled for EB/BRT and 20 were scheduled for BRT.

### Study cohort 3: Canadian patients with prostate cancer

A total of 305 newly-diagnosed PC patients were invited to participate in the study, and 138 were interviewed. Four had poor language skills and could not understand the questionnaires so 134 completed the interview. Sixty-eight (51%) were scheduled to have a RP, and 66 (49%) were scheduled for RT, of whom 61 were scheduled to receive EB, and 5 were scheduled for BRT (for baseline description refer to Tables 1, 2, 3, 4). Among the RT patients, the 5 patients who were scheduled to receive BRT had significantly lower maximum pre-treatment PSA (4.3 ± 2.7 ng/ml vs. 15.1 ± 14.9; p < 0.001), and were all stage T1–T2, but were not significantly different in age (BRT: 65.8 ± 5.8 years, EB: 68.0 ± 6.3 years). In addition, 30 of the RT patients (45%, including one BRT patient) had started ADT by the time of the baseline interview, compared to only 5 (7%) of the RP patients.

### PORPUS-P

Table 5 shows the PORPUS-P scores for the German reference men and each treatment and age group of the German and Canadian PC patients. PORPUS-P scores can range from 0–100; in all subgroups mean scores were in the upper quarter of the scale (indicating high QoL) with the exception of Canadian RT patients aged 50–54 years (n = 2). Means and medians of the subgroups are very similar and indicate a slight right-sided distribution (PORPUS-P scores of German and Canadian RT patients were normally distributed, while data for RP patients was not; Germany: p = 0.013; Canada: p = 0.028 in Kolmogorov-Smirnov tests). In both the German and Canadian cohorts, PC patients about to receive RT had significantly lower crude PORPUS-P scores than those who were about to receive RP (in German data: p = 0.005, in Canadian data: p = 0.001). The age-adjusted means differed slightly between German RT and RP patients and differed in a clinically relevant way (difference of 10.3 points) between Canadian RT and RP patients.

**Table 5:** Scores of PORPUS-P by age categories and type of therapy to be received (Mean ± SD, Median, 5^th ^and 95^th ^percentiles)

	German reference men [n = 988]	German PC patients with scheduled radiotherapy [n = 121]	German PC patients with scheduled radical prostatectomy [n = 371]	Canadian PC patients with scheduled radiotherapy [n = 66]	Canadian PC patients with scheduled radical prostatectomy [n = 68]
Age category [years]					
45–49	n = 253		n = 13	n = 1	n = 1
	87.0 ± 9.70	-	84.8 ± 8.80	83.0	84.2
	88.3 (66.8/100)	-	88.3 (66.8/97.5)	-	-

50–54	n = 239	n = 2	n = 31	n = 2	n = 14
	85.2 ± 11.7	77.8 ± 8.61	82.3 ± 11.9	65.8 ± 13.0	92.1 ± 4.4
	83.3 (61.7/100)	77.8 (71.7/83.8)	83.3 (58.2/100)	65.8 (56.7/75.0)	91.7 (85.8/100)

55–59	n = 162	n = 10	n = 57	n = 4	n = 18
	83.1 ± 12.0	77.1 ± 16.2	81.3 ± 12.5	84.8 ± 5.1	86.5 ± 10.1
	86.3 (62.2/97.5)	77.8 (42.7/100)	84.2 (57.4/95.2)	82.4 (82.2/92.5)	87.1 (69.2/100)

60–64	n = 166	n = 26	n = 117	n = 9	n = 17
	80.9 ± 12.1	77.6 ± 12.7	82.0 ± 11.5	80.3 ± 17.4	85.5 ± 8.2
	84.0 (57.2/97.5)	78.5 (53.9/98.3)	83.3 (59.2/98.2)	85.0 (46.3/100)	86.7 (71.3/97.5)

65–69	n = 127	n = 43	n = 115	n = 21	n = 11
	79.5 ± 13.4	76.9 ± 12.5	79.8 ± 14.1	77.9 ± 10.7	78.5 ± 12.5
	80.8 (57.4/97.5)	76.7 (52.8/94.8)	82.7 (52.3/98.0)	78.8 (65.3/95.0)	79.2 (55.8/95.0)

70+	n = 41	n = 40	n = 38	n = 29	n = 7
	76.7 ± 15.5	77.2 ± 12.4	76.8 ± 10.7	77.0 ± 10.6	78.8 ± 9.1
	78.8 (49.1/97.5)	80.4 (52.2/92.5)	77.8 (56.5/95.0)	75.0 (62.5/92.5)	77.5 (67.2/92.5)

Total (*Crude values*)	*83.5 ± 12.1*	*77.2 ± 12.6*	*80.8 ± 12.4*	*78.0 ± 11.6*	*85.3 ± 9.9*
	*86.1 (59.8/97.5)*	*79.2 (53.5/94.4)*	*83.0 (58.0/97.5)*	*78.8 (62.5/97.5)*	*87.5 (69.2/97.5)*

*Age-adjusted means (norm: ref. pop.; 50 and older)*	*82.3*	*77.4*	*81.3*	*76.0*	*86.3*

ADT influenced (crude) PORPUS-P-scorings of all Canadian patients (with ADT: 74.0 ± 10.4, without ADT: 84.4 ± 10.4; p < 0.001), of all German patients (with ADT: 76.5 ± 12.8, without ADT: 80.5 ± 12.4; p = 0.029), and of all Canadian RT patients (with ADT: 72.5 ± 10.2, without ADT: 82.5 ± 10.8, p < 0.001), but not of Canadian RP patients, German RT and RP patients.

Furthermore, some very low to low but statistically significant inverse associations were observed between age and PORPUS-P scores in the reference men (r = -0.232, p = 0.003), German patients (r = -0.133, p = 0.003) and Canadian patients (r = -0.375, p < 0.001) and in the subgroups of German and Canadian RP patients (r = -0.121, p = 0.019, and r = -0.413, p = 0.005) respectively, but not for RT patients.

**Figure 1:**
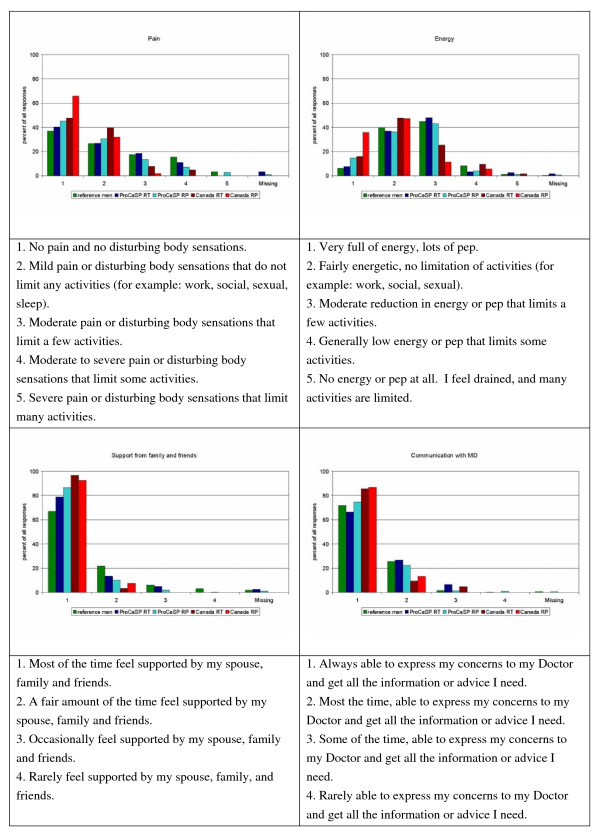
**Distribution of answers for the single items pain, energy, support, and communication (in %)**.

**Figure 2:**
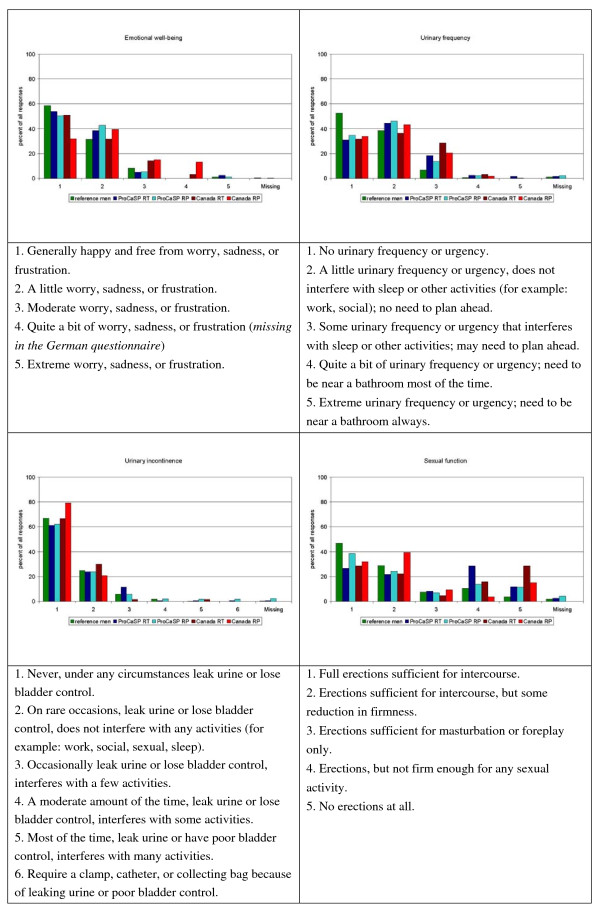
**Distribution of answers for the single items emotional well-being, urinary frequency, urinary incontinence, and sexual function (in %)**.

**Figure 3:**
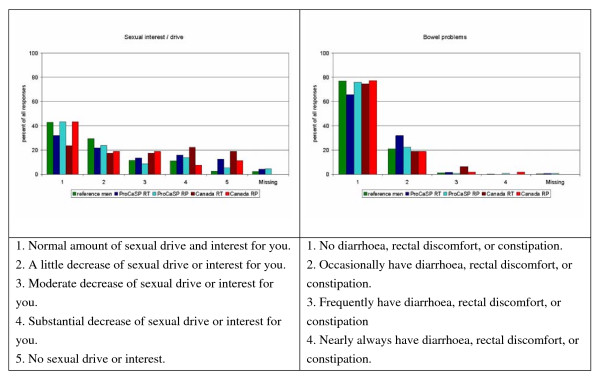
**Distribution of answers for the single items sexual interest and bowel problems (in %)**.

Figures 1, 2, 3 show the distribution of responses for the reference men and PC patients (aged 55 years or more). About 63% of the German patients, and 67% of the Canadian patients reported problems related to urinary frequency or urgency (level 2–5; Figure 2), 49% (German) and 27.6% (Canadian) indicated moderate to low energy (level 3–5), and 54.5% (German) and 44% (Canadian) patients reported mild to severe pain (Figure 1). Bowel problems were reported by 26.9% of the German patients and 23% of the Canadians (Figure 3). Approximately 46% of the reference men reported problems related to urinary frequency or urgency (Figure 2). Energy was considered to be moderate or low by 54.3%, mild to severe pain was reported by 62.9% (Figure 1), and bowel problems were present in 23% of the reference men (Figure 3).

In the German studies, up to two missing values were allowed on the PORPUS. The Canadian study was conducted by personal interview, so all patients answered all PORPUS items. As shown in figure 2 in German men aged 55 years or more the items concerning sexual function and sexual interest/drive had the highest proportions of missing values (patients: 3.8% and 4.5%; reference population: 1.8% and 2.4%).

## Discussion

In this paper, we are reporting on pre-treatment prostate-specific quality of life (QoL) in newly-diagnosed PC patients with a maximum age of 79 years, who were scheduled to receive either RT or RP and in German reference men from the general population.

### Differences between German and Canadian study cohorts

As shown in Tables 1, 2, 3, 4, the German reference men and newly-diagnosed PC patients from Germany and Canada differ in some aspects. In the Canadian study more men were still working than in Germany (Table 2) – although Canadian cancer patients were a little older than German patients (Table 1). In Germany, the usual retirement age of men belonging to the birth cohort 1940 is 65 years. But early retirement, usually at 63 years of age, is possible. In Canada, the usual age for retirement is also 65 years, but many retire as early as 60 years of age, while professionals and self-employed people may work after age 65, either full-time or part-time. Differences between the study groups are also found regarding educational attainment (Table 2). In Germany in 2007, 13% of people over age 45 years had at least 10 years of schooling and another 13% had 12 years [19]. In Canada in 2006, 25% of persons aged 45 to 64 completed high school (12 years of schooling), and 19% had a university degree [20]. Therefore, the Canadian patients in this study were better educated than the Canadian population and than the German cohorts.

From other studies it is known that besides co-morbidity, education and employment affect QoL ratings [21,22]. Because of this and the fact that the two studies were not originally designed for cross-national comparisons, we did not perform statistical tests for differences between QOL scorings of German and Canadian men.

### Differences between patients scheduled to receive radiation therapy or radical prostatectomy

As shown, PC patients from Germany and Canada differ in some aspects which may affect QoL. However, differences also were found between patients scheduled to receive RP and RT (e.g. age (Table 1), education (Table 2), and disease stage (Tables 3, 4)). Other studies, too, reported that patients scheduled for RT (or who have received RT) were older than patients scheduled for RP [23,24], had higher pre-treatment PSA, higher Gleason scores, and more progressed tumours as indicated by T-stage [24,25]. There are two possible reasons for the observed age differences. Older age may be regarded as a risk factor for undesired side effects of surgical procedures, thus RT may be recommended to older men. Secondly, the EAU guideline states that one indication for surgery (RP) is that the expected post-operative survival should be 10 years or more [4]. With increasing age and associated co-morbidities expected survival decreases, therefore older patients might be more likely to receive RT. Furthermore, tumours in younger patients are often more aggressive, so an early excision of the tumour with surgery is indicated.

### Health related quality of life and prostate specific symptoms

We used a prostate-specific tool for assessing QoL: The Patient Oriented Prostate Utility Scale (PORPUS, see additional file 1). This tool is unique in that takes key determinants of QoL such as sexual, urinary and bowel dysfunction into account [11]. Its acceptability, reliability, and validity [11,12,15,16] make it a valuable tool not only in study situations. Due to its shortness, the PORPUS-P can be easily integrated into daily routine in urology practices. The paper-and-pencil form can be completed in approximately 3:30 minutes (pilot study with 20 German men; mean age: 48 years (SD: 15 years); 50% had less than 12 years schooling).

Originally, the PORPUS-P was designed for use in PC patients. But its QoL domains are also relevant among men in the general population, as with increasing age erectile dysfunction [8,9], urinary incontinence [10] and bowel problems are common. Our study indicates that approximately 20% of men in the general population and of PC patients who have not yet been treated with RP or RT, of all ages, have problems in these areas. These observed prevalence rates are a little lower than reported for the general population in Canada and Europe [8-10], furthermore Staff et al. reported pre-treatment prevalence rates for ED of 44% in US patients with localized PC, who were scheduled to receive RT. However, impaired bowel function (8%) or impaired urinary function (18%) was less common in their study than in ours [26]. The authors are only aware of one other study that used PORPUS-P in non-metastatic PC patients and controls. In the study of Joly et al. more PC patients on ADT reported low energy (36%), poor bladder control (47%) and loss of sexual function (95%) than age-matched controls (16%, 34% and 33%, respectively), which could be attributed to the cancer therapy [27].

Problems in other areas of QoL were also reported by our respondents, notably energy level; only 9% of the apparently healthy men, 13% of the German patients, and 26% of the Canadian patients reported feeling "very full of energy, lots of pep", while 6% of the German RT, 5% of the German RP, 11% of the Canadian RT and 6% of the Canadian RP patients reported having generally low energy or pep (level 4) or having no energy at all (level 5).

Monga et al. showed that 8% of veterans (mean age: 67 years) with localized PC experienced fatigue before treatment [28] and Joly et al. reported that 14% of non-metastastic PC patients on ADT and 4% of controls experienced severe fatigue [27]. Schwarz and Hinz assessed QoL with the EORTC QLQ-C30 in the general German population. Of all respondents 10% scored 50 or more points on the fatigue scale (0–100, high scores indicate a high level of symptomatology) [29].

The Canadian and the German version of PORPUS-P have different response schemes for the item "emotional well being". "Quite a bit of worry, sadness, or frustration" was omitted from the German version, so respondents had only 4 levels for this item (see Figure 2). Interestingly, 49.8% of the German patients but only 40% of the Canadians reported being "generally happy and free from worry, sadness, or frustration" (answer category 1). Conversely, only 9.3% of the German patients reported moderate or extreme worry, sadness, or frustration (level 3 and 5), compared with 23% of the Canadian patients who reported moderate, quite a bit, or extreme worry, sadness, or frustration (level 3–5). We do not think that the observed differences are only due to the different response formats in the Canadian and the German versions of the PORPUS. It is possible that "emotional well being" is a different concept in the studied countries.

Comparing the response patterns in Germany and Canada (Figures 1, 2, 3), there are differences between the Canadian English version, as used in Canadian PC patients, and the German version as used in German men (i.e. emotional well being, sexual function), but many of the answer distributions are similar.

The crude PORPUS-P scores differ significantly between RT and RP patients from Germany (mean scores: 77 vs. 81) and Canada (mean scores: 78 vs. 85), respectively. Focusing on the age-specific strata it appears that, in reference men, mean scores decrease by about 4 points per 10 years. Also Schwarz and Hinz found detriments in QoL in the general German population as age increased [29]. In German RP patients, mean PORPUS-P scores decrease after age 60. In Canadian RP patients, mean scores decrease by at least 5 points every 10 years, and these patients have the highest mean scores of all 5 groups up to age 64. There is little or no evidence of age-related decreases in the German or Canadian RT patients.

Based on the works of King et al. [30], Osoba et al. [31], and Ringash et al. [32] a difference of five percent of the scale range can indicate a minimal important difference (i.e. clinically relevant difference). Since the PORPUS-P has a 0 to 100 point scale, we assume a difference of 5 points is clinically relevant (but further studies have to validate this assumption). Thus, we see clinically relevant different PORPUS-P scores between German reference men and German RT patients as well as clinically relevant differences between the PORPUS-P scores of RT and RP patients in both countries. Radiotherapy is often given to PC patients with a higher degree of co-morbidity, which might influence QoL ratings more than the (still untreated) tumour. Other studies showed also that RT patients rate their pre-treatment health related QoL lower than RP patients – this effect is more pronounced when disease specific QoL instruments are used than when using generic instruments [25,33,34].

In our studies rates of ADT were quite similar for Canadian and German RT patients (54 vs. 40%) and RP (2 vs. 7%) patients. Hormone therapy is also known to influence QoL [27] and (as expected) PORPUS-P scorings of all Canadian and of all German patients differed significantly between men with and without ADT. In accordance with our results, Joly et al. [27] showed that non-metastatic PC patients with at least three months of ADT had significantly lower PORPUS-P scores than healthy age-matched control men (median: 71 vs. 86; p < 0.001).

### Missing values

The Canadian data, collected in personal interviews, have no missing data, but the German studies were conducted by mail so there were missing values. It is often assumed that persons are unwilling to answer questions about sexual and urinary function. This assumption is true for our data; 18.1% of the German PC patients had missing data for the item sexual function, 8.7% gave no answer to the item on sexual interest, 6.4% provided no answer regarding urinary leakage, and 5.7% had missing data concerning urinary frequency. In comparison, only 0.3% to 1.7% of the German reference men did not answer these questions.

Furthermore it has been suggested that missing values are more common when problems are present (thus, people are more willing to report the absence of problems) [35]. Under the assumption that missing values indicate a high level of deterioration, the "true" German values might be overestimated by our study samples and differences in QoL ratings between Canada and Germany might be even more marked than in data presented here. Replacing missing values with the level indicating the most severe problem would result in crude PORPUS-P values of 82.6 for the reference men, 76.2 for RT patients and 79.9 for RP patients. Replacing missing values with the level indicating no problem would reveal mean values of 83.3 for the reference men, 77.5 for RT and 81.2 for RP patients.

### Strengths and Limitations

In the present study, we were able to report on three datasets from two countries and conduct a cross-national comparison of men with PC. One of the strengths is the inclusion of nearly 1,000 reference men from the general population, who provided information on prostate specific and general QoL. Unfortunately, it was not possible to give a questionnaire on health status to the reference men, and only 50% of the German PC patients answered a checklist on co-morbidities, while this information is available for patients from Canada. The observed age effect in RP patients may be due to other factors that were not examined in German patients such as co-morbidity. In the Canadian study the Charlson co-morbidity score was computed [14]. Both age and co-morbidity were significantly correlated with PORPUS-P scores (for age and PORPUS-P, r = -0.386, p < 0.001, and for co-morbidity and PORPUS-P, r = -0.206, p = 0.017). But there was no significant relationship between age and Charlson score (r = 0.082, p = 0.34), which might be due to the inhomogeneous distribution of Charlson scores in the group. Furthermore, it might be possible that due to differing exclusion and inclusion criteria for prostate cancer patients in Germany and Canada both study groups may be heterogeneous in characteristics that we did not measure or report here.

More than any other tumour, PC is a neoplasm of the elderly. For example, in Germany, the mean age at diagnosis is approximately 70 years and more than 90% are aged 60 years or older [2]. The median age of our German cancer patients was 64 years. In the German studies elderly and very old patients were not allowed to participate due to reasons of remembrance abilities and capacity. In the Canadian study poor cognitive abilities was an exclusion criterion, and the oldest participant was 79 years. As we have observed inverse relationships between age and QoL scoring in all three studies it might be possible that the reported QoL ratings are too optimistic to be generalized to all prostate cancer patients (including the elderly and the very old patients).

## Conclusion

The PORPUS-P is a short self-administered questionnaire which can be easily integrated into research and medical practice. The German translation of the PORPUS-P appears to be a feasible tool for assessing prostate-specific quality of life. Our data show that patients from Canada and Germany scheduled for RT have lower PORPUS-P scores than patients scheduled for RP and than reference men from Germany – with the exception of the oldest age group included in our studies, in which co-morbidities might have a greater impact on QoL. Differences in quality of life between patients scheduled to receive radiotherapy and radical prostatectomy exist before treatment starts. Therefore pre-treatment (baseline) quality of life measures are seriously needed, when effects of different therapies on quality of life are evaluated.

Although we found a similar response pattern, PC patients scheduled to receive either RT or RP from Canada and Germany rated their pre-treatment quality of life on different levels, which reveals the need for national reference data.

## Competing interests

The authors declare that they have no competing interests.

## Authors' contributions

AW has been involved in drafting and writing the manuscript, has made substantial contributions to statistical analysis (German data) and interpretation of data. VR has made substantial contributions to conception and design of the German studies, acquisition of German data, has been involved in writing the manuscript and interpreting of data. KB has made substantial contributions to conception and design of the Canadian study, acquisition of data, has been involved in writing the manuscript, made substantial contributions to statistical analysis (Canadian data) and interpretation of data. MK has made substantial contributions to conception and design of the Canadian study, acquisition of data, and has been involved in revising the manuscript critically for important intellectual content. TK has made substantial contributions to conception and design of the German studies, and acquisition of German data. AK has been involved in drafting the manuscript and revising it critically for important intellectual content.

## Pre-publication history

The pre-publication history for this paper can be accessed here:

http://www.biomedcentral.com/1471-2407/9/295/prepub

## Supplementary Material

Additional file 1 **German Version of PORPUS-P**. German version of the questionnaire "PORPUS"). Click here for file
