# Supplementary material for: Measuring prostate-specific quality of life in prostate cancer patients scheduled for radiotherapy or radical prostatectomy and reference men in Germany and Canada using the Patient Oriented Prostate Utility Scale-Psychometric (PORPUS-P)
Source: BMC Cancer. 2009 Aug 23;9:295. doi: 10.1186/1471-2407-9-295 (PMC2739225; doi:10.1186/1471-2407-9-295)
Supplement: Additional file 1 — German Version of PORPUS-P. German version of the questionnaire "PORPUS"). [file 1471-2407-9-295-S1.pdf]

Patient Oriented Prostate Cancer Utility Scale (PORPUS) – Deutsche Version 1.1

**I. Schmerzen und störende körperliche Empfindungen**<br>***(wie z. B. Hitzewallungen, schmerzhaftes Anschwellen der Brust, Übelkeit)***<br>Bitte kreuzen Sie die Aussage an, die in den letzten zwei Wochen am ehesten auf Sie zutraf.		
1	☐	Ich habe keine Schmerzen oder störende körperliche Empfindungen.
2	☐	Ich habe geringe Schmerzen oder störende körperliche Empfindungen, die mich aber nicht behindern (bei meiner Arbeit, sozialen oder sexuellen Aktivitäten, Schlaf).
3	☐	Ich habe mäßige Schmerzen oder störende körperliche Empfindungen, die wenige meiner Aktivitäten beeinflussen.
4	☐	Ich habe mäßige Schmerzen oder störende körperliche Empfindungen, die einige meiner Aktivitäten beeinflussen.
5	☐	Ich habe starke Schmerzen oder störende körperliche Empfindungen, die viele meiner Aktivitäten beeinflussen.
**II. Energie**<br>Bitte kreuzen Sie die Aussage an, die in den letzten zwei Wochen am ehesten auf Sie zutraf.		
1	☐	Ich bin voller Energie und Schwung.
2	☐	Ich bin eher Energie geladen, habe keine Einschränkungen bei Aktivitäten (wie z. B. bei Arbeit, sozialen oder sexuellen Aktivitäten, Schlaf).
3	☐	Ich verspüre eine mäßige Beeinträchtigung von Energie und Schwung, die einige meiner Aktivitäten beeinflussen.
4	☐	Ich habe generell wenig Energie und Schwung, was einige meiner Aktivitäten beeinträchtigt.
5	☐	Ich habe keine Energie und keinen Schwung, fühle mich erschöpft und viele meiner Aktivitäten sind beeinträchtigt.
**III. Unterstützung von Familie und Freunden**<br>Bitte kreuzen Sie die Aussage an, die in den letzten zwei Wochen am ehesten auf Sie zutraf.		
1	☐	Ich fühle mich die meiste Zeit unterstützt von meinem Partner, der Familie und Freunden.
2	☐	Ich fühle mich einen großen Teil der Zeit unterstützt von meinem Partner, der Familie und Freunden.
3	☐	Ich fühle mich gelegentlich unterstützt von meinem Partner, der Familie und Freunden.
4	☐	Ich fühle mich kaum unterstützt von meinem Partner, der Familie und Freunden.

IV. Kommunikation mit dem Arzt

(Behandelnder Arzt, kann ein Spezialist oder auch der Hausarzt sein.)

Bitte kreuzen Sie die Aussage an, die während der letzten zwei Behandlungen am ehesten auf Sie zutraf.

1	☐	Ich bin jederzeit in der Lage, meine Bedürfnisse meinem Arzt gegenüber auszudrücken und bekomme alle Informationen und Ratschläge, die ich brauche.
2	☐	Meistens bin ich in der Lage, meine Bedürfnisse meinem Arzt gegenüber auszudrücken, um alle Informationen und Ratschläge, die ich brauche, zu bekommen.
3	☐	Manchmal bin ich in der Lage, meine Bedürfnisse meinem Arzt gegenüber auszudrücken, um alle Informationen und Ratschläge, die ich brauche, zu bekommen.
4	☐	Ich bin selten in der Lage, meine Bedürfnisse meinem Arzt gegenüber auszudrücken, um alle Informationen und Ratschläge, die ich brauche, zu bekommen.

V. Emotionales Wohlbefinden

Bitte kreuzen Sie die Aussage an, die in den letzten zwei Wochen am ehesten auf Sie zutraf.

1	☐	Ich bin überwiegend glücklich, nicht bedrückt, traurig oder unzufrieden.
2	☐	Ich bin ein wenig bedrückt, traurig oder unzufrieden.
3	☐	Ich bin mäßig bedrückt, traurig oder unzufrieden.
4	☐	Ich bin sehr bedrückt, traurig oder unzufrieden.

VI. Häufiges Wasserlassen / nicht unterdrückbarer Harndrang

(Häufiges Wasserlassen während des Tages oder der Nacht, Harndrang (=Schwierigkeiten das Wasserlassen hinaus zu zögern; Unfähigkeit de Urin länger zu halten).)

Bitte kreuzen Sie die Aussage an, die in den letzten zwei Wochen am ehesten auf Sie zutraf.

1	☐	Ich habe keine Beschwerden.
2	☐	Ich habe geringe Beschwerden, ich muss nicht im Voraus planen.
3	☐	Ich habe einige Beschwerden, diese beeinträchtigen den Schlaf und andere Aktivitäten, ich muss weit im Voraus planen.
4	☐	Ich habe starke Beschwerden, ich muss mich meistens in der Nähe der Toilette aufhalten.
5	☐	Ich habe sehr starke Beschwerden, ich muss mich ständig in der Nähe der Toilette aufhalten.

VII. Kontinenz

(Unfreiwilliger Harnabgang, Unvermögen, den Harn zurückzuhalten.)

Bitte kreuzen Sie die Aussage an, die in den letzten zwei Wochen am ehesten auf Sie zutraf.

1	☐	Ich habe niemals, unter keinen Umständen unfreiwilligen Harnabgang.
2	☐	Ich habe selten unfreiwilligen Harnabgang, was aber meine Arbeit, soziale und sexuelle Aktivitäten oder meinen Schlaf nicht beeinträchtigt.
3	☐	Ich habe gelegentlich unfreiwilligen Harnabgang, das beeinträchtigt wenige meiner Aktivitäten.
4	☐	Ich habe mäßig unfreiwilligen Harnabgang, das beeinträchtigt einige meiner Aktivitäten.
5	☐	Ich habe meistens unfreiwilligen Harnabgang, das beeinträchtigt viele meiner Aktivitäten.
6	☐	Ich benötige eine Klemme, einen Katheter oder einen Urinbeutel.

VIII. Sexuelle Funktion

(Probleme eine Erektion zu erreichen und zu halten; mit oder ohne Hilfsmittel wie beispielsweise Injektion oder Prothese.)

Bitte kreuzen Sie die Aussage an, die in den letzten zwei Wochen am ehesten auf Sie zutraf.

1	☐	Ich habe vollständige Erektionen, die ausreichend steif für den Geschlechtverkehr sind.
2	☐	Ich habe Erektionen, die ausreichend steif für den Geschlechtverkehr sind, diese jedoch mit Einschränkungen.
3	☐	Ich habe Erektionen, die ausreichend steif sind für Masturbation oder Vorspiele.
4	☐	Ich habe Erektionen, die nicht ausreichend steif für irgendeine sexuelle Aktivität sind.
5	☐	Ich habe keine Erektionen.

IX. Sexuelles Verlangen und Interesse

Bitte kreuzen Sie die Aussage an, die in den letzten zwei Wochen am ehesten auf Sie zutraf.

1	☐	Mein sexuelles Verlagen und Interesse sind normal für mich.
2	☐	Mein sexuelles Verlagen und Interesse haben geringfügig nachgelassen.
3	☐	Mein sexuelles Verlagen und Interesse haben mäßig nachgelassen.
4	☐	Mein sexuelles Verlagen und Interesse haben wesentlich nachgelassen.
5	☐	Ich verspüre kein sexuelles Verlangen oder Lust.

X. Verdauungsbeschwerden

(Verstopfung, Durchfall, Darmbeschwerden wie Schmerzen, Brennen oder Irritation)

Bitte kreuzen Sie die Aussage an, die in den letzten zwei Wochen am ehesten auf Sie zutraf.

1	☐	Ich habe keinen Durchfall, keine Darmbeschwerden oder Verstopfung.
2	☐	Ich habe gelegentlich Durchfall, Darmbeschwerden oder Verstopfung.
3	☐	Ich habe regelmäßig Durchfall, Darmbeschwerden oder Verstopfung.
4	☐	Ich habe ständig/fast immer Durchfall, Darmbeschwerden oder Verstopfung.
